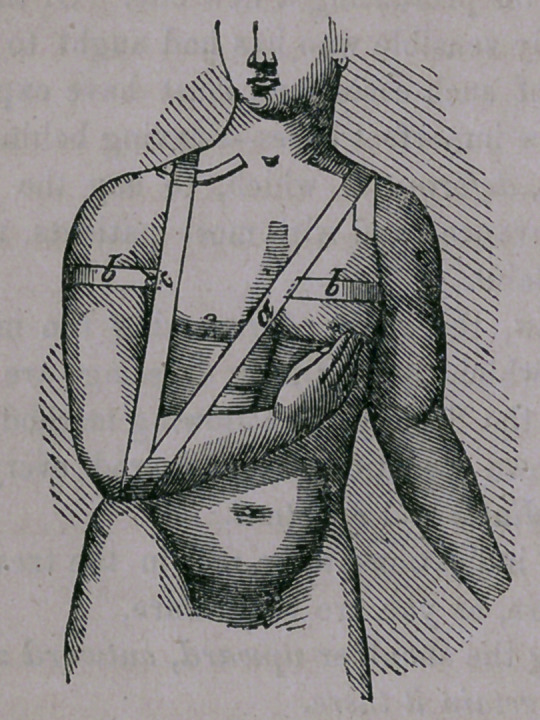# A New Mode of Dressing Fractured Clavicle

**Published:** 1858-10

**Authors:** J. W. Freer

**Affiliations:** Prof. of Anatomy in Rush Medical College, Surgeon to the Mercy Hospital, Etc., Etc., Etc.


ARTICLE VII.

### A NEW MODE OF DRESSING FRACTURED CLAVICLE.

BY J. W. FREER, M. D., PROF. OF ANATOMY IN RUSH MEDICAL COLLEGE, SURGEON TO THE MERCY HOSPITAL, ETC., ETC., ETC.

Among the many and varied appliances used in surgery in the dressing of fractured bones, few have presented to the surgeon greater difficulties in their application, so as to meet the several indications present, or have more frequently been the occasion of provoking annoyance both to himself and patient, than the ordinary dressings for fractured clavicle. Indeed, so invariably has this been felt, that many have been the plans and modes suggested, each intended to obviate the objections which were justly chargeable to all the others.

It is to this inefficiency and trouble so generally experienced in the application and subsequent keeping in place of the ordinary dressings (for fractured clavicle), that I must refer you for my apology for presenting a new one. Of these evils every one must be duly sensible who has had aught to do in the care and treatment of such cases, and must have experienced, with regret, not a few imperfect cures—leaving behind them, in very many instances, *deformities*, which, to say the least, are unsightly in appearance, and with most patients, not unfrequent causes of complaint.

With the view, therefore, of removing the many objections and results, to which the ordinary dressings are liable, I herewith submit to the Society the following method of treatment, which in my own practice has answered every expectation, being *simple*, *reliable* and *effectual*.

The different indications to be met in the treatment of fractured clavicle are, as you are well aware,

1*st*, To bring the shoulder *upward*, *outward* and *backward*.

And 2*d*, To *retain it there*.

These several indications are fulfilled by the use of *adhesive straps*, applied in the following manner—to wit:

1*st*, A strip of adhesive plaster, of two and a-half or three inches in width, and of sufficient length to extend from the under surface of the forearm, near the elbow of the affected side—to the shoulder of the opposite side (see *a* in the accompanying diagram),—the strap being applied about its *middle* to the forearm, and passing each *end*, one in *front* and the other *behind*, and crossing them upon the shoulder; the ends being permitted to extend downward, and lapping, one upon the *breast* and the other upon the *back*—drawing it sufficiently tight to bring the elbow firmly to the *side* and elevate the shoulder—a *pad* (*d*) having previously been placed in the *axilla*, for the purpose of carrying the shoulder outward.

2*d*, A *strap* of like width, passed around the arm of the affected side, at the axilla, (see *b*) and carried *across* the back and *under* the arm of the opposite side and lapping upon the breast, drawn sufficiently tight to bring the shoulder *backward* to the required extent.

3*d*, The hand may be supported by placing it in a silk handkerchief attached to a loop of adhesive plaster passing over the *sound* shoulder.

If from any cause it be desirable at any time to make compression over the fractured ends of the bone, it may be readily done by passing a strap of adhesive plaster from the forearm of the affected side *over* the affected shoulder. (See *c*.)

The following cases were treated after the manner described:

CASE 1.—J. Hubbard. Clavicle fractured at the *outer* third. Considerable depression of the shoulder. Dressing by adhesive straps, and removal at the end of twenty-one days. No interference was required during the process of cure.

CASE 2.—J. S., aged about fifty. Comminuted fracture at the middle, occasioned by direct violence. Three days had elapsed since the injury, when he came under my care. This case was treated like the preceding one, with the addition of a strap of adhesive plaster extending over the fragments (see *c*), in order to produce slight compression. The patient lived some distance in the country, and consequently I did not see him again until six weeks had elapsed. He had removed the dressings on his own responsibility at the end of twenty-five days. The patient informed me that the dressings had not been interfered with or disturbed up to the time of their removal.

The cure was perfect, as far as the symmetry of the shoulder was concerned; but as usually follows in comminuted fractures, a *callus* was remaining, though of moderate dimensions.

CASE 3.—James M., aged ——, suffered a fracture of the clavicle near the *outer* third. Treatment in the same manner.

The patient was informed that he need not present himself again until the end of twenty days, at which time I removed the dressings.

Cure perfect.

## Figures and Tables

**Figure f1:**